# Pediatric Hematopoietic Stem Cell Transplantation in Lithuania – 20 Years of Progress through Collaboration

**DOI:** 10.15388/Amed.Supp.2022.292.2

**Published:** 2022-06-29

**Authors:** Jelena Rascon, Goda Elizabeta Vaitkevičienė, Sonata Šaulytė Trakymienė, Ramunė Pasaulienė

**Affiliations:** The Organizing Committee; The Organizing Committee; The Organizing Committee; The Organizing Committee

**Keywords:** hematopoietic stem cell transplantation, children, Lithuania, survival, transplant-related mortality

Hematopoietic stem cell transplantation (HSCT) is a complex procedure that is curative for several fatal pediatric malignancies and non-malignant diseases. Despite its complexity, potential toxicity, and high costs HSCT has become a standard procedure worldwide for several decades. Pediatric HSCT programs encounter several specific challenges. The rarity and heterogeneity of primary diseases, result in an almost 10-fold inferior number of pediatric HSCT as compared to adults. In contrast to the adult programs, where autologous HSCT is more common, allogeneic HSCT (that is more complex) prevails in pediatric setting which is underpinned by a higher number of inborn disorders transplanted in early childhood.

In Lithuania, the pediatric HSCT program (EBMT CIC* 508) was launched at Vilnius University

Hospital Santaros Klinikos in February 2002. Currently, this is the only specialized pediatric HSCT center in Lithuania and in the Baltic countries. Since 2011 it is a reference center for Latvian children who need autologous or allogeneic transplantation.

Here we summarize conference proceedings presented at the scientific event “Pediatric hematopoietic stem cell transplantation in Lithuania – 20 years of progress through collaboration”. The meeting held on September 22-23, 2022, in Vilnius and aimed at commemorating 20 years of the launch of the pediatric transplant program in Lithuania. The event pursued sharing the experience in the field of pediatric HSCT in the Baltic countries. Given a very small population in all three Baltic countries, Lithuania, Latvia, and Estonia face an additional challenge in maintaining sufficient transplant volume and gaining experience. Several distinguished speakers from USA, Denmark, Italy, Germany, Spain, UK and Ukraine shared their expertise in the field and emphasized the crucial role of national and international collaboration to achieve progress in the management of this very rare and complex procedure that offers cure for otherwise fatal pediatric conditions.

## Footnotes

* EBMT CIC – European Society for Blood and Marrow Transplantation Center Identification Code
