# Supplementary material for: Pediatric Hematopoietic Stem Cell Transplantation in Lithuania – 20 Years of Progress through Collaboration
Source: Acta Med Litu. 2022 Jun 29;29(2):1–17. doi: 10.15388/Amed.Supp.2022.292.2 (PMC9799012; doi:10.15388/Amed.Supp.2022.292.2)
Supplement: Translation

# Translation: First Experience with Dinutuximab in Children with High-Risk Neuroblastoma

**Authors:** Karolina Tumelienė, Indrė Tamulienė, Jelena Rascon

**Affiliations:** 1 Clinic of Pediatrics, Institute of Clinical Medicine, Faculty of Medicine, Vilnius University, Vilnius, Lithuania; 2 Center for Pediatric Oncology and Hematology, Vilnius University Hospital Santaros Clinics, Vilnius, Lithuania

**Keywords:** dinutuximab, GD2, immunotherapy, monoclonal antibody, neuroblastoma

## Abstract

**Background:**

Neuroblastoma (NB) is the most common extracranial solid tumor in childhood. High-risk NB still has a poor prognosis and requires complex aggressive therapy. For a decade, immunotherapy with dinutuximab – anti GD2 chimeric monoclonal antibody – was integrated into treatment protocols as a routine part of the treatment. However, drug administration is related to potentially severe adverse effects. Thus, its administration is recommended in experienced centers. We aim to describe the first Lithuanian experience with dinutuximab.

**Methods:**

We performed a retrospective analysis of patients with high-risk NB treated with dinutuximab at our institution in 2020-2022. Toxicity and treatment outcomes were evaluated. The data were retrieved from electronic and paper records.

**Results:**

in 2020-2022, four patients were treated with dinutuximab. Totally, 20 cycles were delivered, 5 cycles in each patient. One patient received dinutuximab as the first-line treatment. In 3 children dinutuximab was administered as second-line therapy with a median of 18 months (range 9-21) after relapse. For the majority of patients, the treatment tolerance was acceptable. The main adverse events were fever (n = 4), vision impairment (n = 1) and capillary leak syndrome (n = 1). The adverse events were 1-2 degrees, so all patients completed the treatment. At the time of analysis, two patients remain in complete remission, one patient achieved stable disease and one patient died because of disease progression. Five-year overall survival was 66.7%

**Conclusions:**

Immunotherapy is currently the standard for first-line maintenance treatment of high-risk neuroblastoma. Dinutuximab administration is associated with a risk of severe adverse events, so the treatment should be performed in an experienced pediatric oncology center.

# Translation: Invasive Fungal Infection in T-cell Acute Leukemia Patient

**Authors:** Brigita Valašinaitė, Sigita Stankevičienė, Goda Elizabeta Vaitkevičienė, Jelena Rascon

**Affiliations:** 1 Clinic of Children‘s Diseases, Institute of Clinical Medicine, Vilnius University Faculty of Medicine, Vilnius, Lithuania; 2 Center for Pediatric Oncology and Hematology, Vilnius University Hospital Santaros Klinikos, Vilnius, Lithuania

## Abstract

**Introduction:**

Early recognition and immediate antifungal treatment are crucial for the control of invasive fungal infection (IFI). IFI is an important cause of morbidity and mortality in immunocompromised patients: it has an in-hospital mortality rate of 15.8 percent of pediatric patients with candidemia and 18 percent of children with invasive aspergillosis. We aim to present a case of IFI.

**Case presentation:**

A 3-year-old boy was repeatedly treated at Vilnius University Hospital Santaros Klinikos for recurrent viral or bacterial infections with unexplained pancytopenia and occasional lymph node enlargement. Bone marrow showed increased lymphoblasts, however, not reaching the criteria for leukemia diagnosis. At 4 years of age, the boy was diagnosed with early T-precursor acute leukemia (ETP). Chemotherapy was initiated according to the ALLTogether protocol, remission and negative MRD were achieved. At the end of induction, neutropenia and systemic candidemia developed (*Candida tropicalis*). At the beginning of Consolidation 1 phase, the patient developed fever and polymorphic skin rash, which later developed into skin pustules, which ruptured occasionally. The skin biopsy showed chronic inflammation with PAS/Groccot positive fungi. CBC showed pancytopenia, later developed to neutrophilosis. Regardless of sufficient leuko- and neutropoiesis, a disseminated IFI with multiple infiltrates in liver, spleen and kidney were detected. The patient was treated with different combinations of five antifungal agents. Multiple blood cultures were repeated, no fungi growth was identified. There is a suspicion of an underlying genetic syndrome that causes prolonged pancytopenia and final transformation to an ALL.

**Conclusions:**

In this case an IFI – systemic candidemia – is resilient to management despite adequate neutrophil production and antifungal therapy. An atypical toxicity profile should raise a concern about an underlying genetic disorder.

# Translation: Allogeneic HSCT for Pediatric Acute Myeloid Leukemia in Lithuania

**Authors:** Kajus Merkevičius, Ramunė Pasaulienė, Igne Kairienė, Goda Elizabeta Vaitkevičienė, Vilma Rutkauskaitė, Ramunė Vaišnorė, Audronė Jakaitienė, Jelena Rascon

**Affiliations:** 1 Clinic of Pediatrics, Institute of Clinical Medicine, Faculty of Medicine, Vilnius University, Vilnius, Lithuania; 2 Center for Pediatric Oncology and Hematology, Vilnius University Hospital Santaros Klinikos, Vilnius, Lithuania; 3 Institute of Data Science and Digital Technologies, Vilnius University, Lithuania; 4 Faculty of Medicine, Vilnius University, Vilnius, Lithuania

## Abstract

**Background.:**

Allogeneic hematopoietic stem cell transplantation (aHSCT) is the only curative treatment for childhood high-risk, primary refractory, or relapsed acute myeloid leukemia (AML). In Lithuania AML-BFM protocol was used to treat pediatric AML in 2002-2013. Since 2014 the first- line treatment switched to the NOPHO-DBH AML protocol. The risk stratification, indications for aHSCT differed between the protocols.

**Aim.:**

To evaluate aHSCT outcomes in children transplanted for AML at our institution in 2002-2021.

**Methods.:**

A retrospective analysis of medical records was performed. The treatment outcomes were compared between 2002-2011 (1^st^ period) and 2012-2021 (2^nd^ period).

**Results.:**

AML was diagnosed in 81 children in 2002-2021 (52 cases in 1^st^ period, 29 cases in 2^nd^period). In the 1^st^ and 2^nd^ periods, out of all AML patients, 17.3% (n = 9) and 58.6% (n = 17; including 4 Latvian children) were treated with an aHSCT, respectively. Analysis of aHSCT outcomes revealed no difference in transplant-related mortality (TRM) between the 1^st^ and the 2^nd^ periods: the probability of TRM at 100 days was 0.220 (95 %CI [0.028, 0.530]) vs 0.120 (95 %CI [0.018, 0.320]), and at 5 years 0.615 (95% CI [0.280, 0.831]) vs 0.087 (95% CI [0.014, 0.246]), respectively (p =0.1732). Outcomes evaluated at 5 years after aHSCT showed a statistically significant improvement from 1^st^ to 2^nd^ periods: the cumulative incidence of relapse decreased from 0.486 (95% CI [0.073, 0.820]) to 0.067 (95% CI [0.004, 0.269]), p = 0.0402; the probability of event-free survival increased from 0.333 (95% CI [0.132, 0.840]) to 0.765 (95% CI [0.587, 0.995]), p = 0.0462, as the probability of overall survival did: 0.333 (95% CI [0.132, 0.840]) vs 0.765 (95% CI [0.587, 0.995]), p = 0.0488, respectively.

**Conclusions.:**

The most analysed outcomes of aHSCT performed for pediatric AML improved significantly over two decades, except for early TRM. The results should be interpreted with caution due to the small sample size.

# Translation: A Challenging Case of Acute Leukemia Complicated by a Ruptured Aortic Root Caused by Catheter-Related Infective Endocarditis

**Authors:** Milita Stuopelytė, Odeta Kinčinienė, Eitautė Jakutienė, Vilma Rutkauskaitė, Rūta Gegieckienė, Karolis Jonas, Rita Sudikienė, Virgilijus Tarutis, Sonata Šaulytė-Trakymienė

**Affiliations:** 1 Clinic of Children‘s Diseases, Institute of Clinical Medicine, Faculty of Medicine, Vilnius University, Vilnius, Lithuania; 2 Vilnius University Hospital Santaros Clinics, Vilnius, Lithuania; 3 Center of Cardiothoracic Surgery, Clinic of Cardiovascular Diseases, Institute of Clinical Medicine, Faculty of Medicine, Vilnius University, Vilnius, Lithuania

## Abstract

**Background.:**

Bloodstream infection is one of the most important causes of morbidity and mortaity among patients with acute leukemia. The risk of infection is increased due to indwelling venous catheters, long-term hospitalization, multidrug-resistant microorganisms, and other predisposing factors. Nevertheless, native valve infective endocarditis (IE) is a rare complication.

**Aim.:**

To report a first complicated case of IE due to *S. aureus* in a patient with acute leukemia.

**Case presentation.:**

A 21-month-old boy presented with fever, sleep deprivation and appetite loss. Laboratory tests indicated: WBC, 18,2*10^9/l, NEU, 0,77*10^9/l; HGB 68 g/l; platelets, 26 *10^9/l, 56% blasts on blood smear. The diagnosis of B-precursor acute lymphoblastic leukemia (ALL) with high hyperdiploidy was based on bone marrow **Results.** After successful induction according to the Nopho ALLTogether protocol, the patient was stratified to the final intermediate-high risk group because of 1,3% MRD by flow cytometry. During consolidation, S. *aureus* caused sepsis occurred twice. After the second sepsis, the central venous line (CVL) was removed. However, the persistence of S. *aureus* was confirmed from blood culture ten days later. Physical examination revealed a continuous murmur and tachycardia. Two-dimensional transthoracic echocardiography (TTE) showed echogenic structures on the aortic valve leaflets. The presence of new moderate-severe aortic valve regurgitation and signs of pericardial effusion were suspected. IE was confirmed and treated according to microbiology test **Results.** Dilatation and dysfunction of the left ventricle progressed during the next few weeks. Aortic root rupture with penetration into the interventricular septum was suspected by TTE and confirmed by computed tomography. Consequently, the patient underwent successful surgical repair of the aortic root and the aortic valve. Intravenous antibiotics were administered for 6 weeks. There were no complications during follow-up. Anti-leukemic treatment was restarted after four weeks with negative flow cytometry for MRD.

**Conclusions:**

Infective endocarditis complicated by an aortic root rupture that penetrates to the interventricular septum is a rarity among children. However, catheter-related bloodstream infections might cause infective endocarditis, especially in patients with underlying conditions like acute lymphoblastic leukemia.

# Translation: Outcomes of Childhood Relapsed Acute Lymphoblastic Leukemia and the Role of Hematopoietic Stem Cell Transplantation in Lithuania

**Authors:** Goda Vaitkevičienė, Ramunė Pasaulienė, Jelena Rascon, Vilma Rutkauskaitė

**Affiliations:** 1 Centre for Pediatric Oncology and Hematology, Vilnius University Hospital Santaros Klinikos, Vilnius, Lithuania; 2 Vilnius University, Faculty of Medicine, Lithuania

## Abstract

**Background.:**

Long-term survival rates of childhood acute lymphoblastic leukemia (ALL) approach 90%, however, still a proportion of children will suffer from a relapse. Only around 50% of children with a first relapse survive long-term, and the outcome is much worse with a second or later relapse.

**Aim of the study.:**

To analyze the incidence and treatment results of childhood ALL relapses in Lithuania throughout different time periods.

**Methods.**

Retrospective analysis of data of 98 children (out of 105) with relapsed T- or B-cell precursor ALL treated in Lithuania during the four time periods: 1992-1996 (N=38), 1997-2002 (N=33), 2003-2008 (N=16) and 2009-2018 (N=18) for whom the data was available.

**Results.:**

Median follow-up (quartiles) for four periods was 18.2 (17.9-18.2), 15.1 (9.2-16.8), 12.6 (10.9-14.5) and 3.9 (2.0-6.1) years, respectively. Overall survival (OS) improved over periods, with 3-year OS being 30±7%, 28±8%, 44±12%, 50±12% respectively, although non-significantly. Twenty-four patients received allogeneic hematopoietic stem cell transplantation (HSCT) for very early (n=7), early (n=5) or late (n=12) relapse. Survival improvement was seen over the four time periods, 3-year OS being 0%, 43±19%, 50±16% and 89±11%, respectively. Busulfan, cyclophosphamide and etoposide were used for conditioning for 20/24 patients (83.3%).

**Conclusions.:**

Cure for children with relapsed ALL remains challenging although reaching the rates reported by the large international groups during the last decade. Improving laboratory possibilities allowing more accurate MRD assessment, stratification of relapsed patients to alloHSCT versus chemotherapy and identification of the best possible stem cell donor as well as increasing experience of our bone marrow transplantation center can be named as one of the reasons for improving alloHSCT results which in turn add to the improvement of survival rates.

# Translation: Early Secondary Leukemia after a Relapsed Wilms’ Tumor

**Authors:** Milita Stuopelytė, Indrė Tamulienė, Rolanda Nemanienė, Jelena Rascon

**Affiliations:** 1 Vilnius University, Faculty of Medicine, Lithuania; 2 Center for Paediatric Oncology and Haematology, Vilnius University Hospital Santaros Klinikos, Vilnius, Lithuania

## Abstract

**Background.:**

Wilms’ tumor (WT) is the most prevalent childhood renal neoplasm. Although overall cure rate is high, relapse occurs in 15% of patients. Another concern is the emergence of treatment related secondary malignancies of which acute myeloid leukaemia represents 15-20%. It usually occurs 3-10 years after initial therapy and is particularly devastating.

**Case.:**

A 6 year old girl with metastatic WT (lungs, nodules size >5 mm) was treated in our hospital. The patient has a healthy monozygotic twin. Preoperative AVD chemotherapy was given by Umbrella SIOP-RTSG 2016 protocol. Following 6 weeks of preoperative chemotherapy, metastasis were absent in chest CT. Postoperative treatment after nephrectomy was given according to local stage (III) and histology (intermediate risk tumor - mixed type nephroblastoma): regimen AVD250 and flank irradiation.

7 months from nephrectomy first early lung relapse was confirmed. Histology assessment showed blastema with focal anaplasia. Complete remission achieved after BB risk group treatment by Umbrella protocol: chemotherapy ICE/CyCE, surgery, HD-LPAM with autologous PBSC rescue and pulmonary RT. Early relapse and blastema component were unexpected for us therefore primary tumor histology reassessment was done.

Second lung relapse emerged 5 months after end of treatment. After nonradical excision of tumor masses histology showed diffuse anaplasia. Salvage VIT therapy was started. 8 months later a very early secondary acute monoblastic leukaemia arose (M5 according to FAB). Treatment was initiated by NOPHO-DBH 2012 protocol. Dynamics in bone marrow aspirate after 3 chemotherapy courses was positive. Our patient was in the allogeneic bone marrow transplantation waiting list. However, chest CT revealed lung metastasis progression. Sadly exhaustion of all active treatment options was concluded. Currently the patient receives metronomic therapy.

# Translation: Blinatumomab in Pediatric ALL: a Lithuanian Case Series

**Authors:** Kajus Merkevičius, Vilma Rutkauskaitė, Sonata Šaulytė-Trakymienė

**Affiliations:** 1 Clinic of Pediatrics, Institute of Clinical Medicine, Faculty of Medicine, Vilnius University, Vilnius, Lithuania; 2 Center for Pediatric Oncology and Hematology, Vilnius University Hospital Santaros Klinikos, Vilnius, Lithuania

## Abstract

**Background.:**

Pediatric refractory / relapsed acute lymphoblastic leukemia (R/R-ALL) has a 5% long term survival rate when applying cytotoxic chemotherapy, hematopoietic stem cell transplantation (HSCT). Blinatumomab, a bispecific antibody linking CD19, CD3 cells, may induce beneficial effect before HSCT and has successfully been used as a frontline therapy for high risk (HR) patients [1,2].

**Aim.:**

To present 5 R/R-ALL cases treated with Blinatumomab.

**Methods.:**

Retrospective case series.

**Results.:**

A 4-month girl (pre-B, 11q23 MLL (KMT2A), CNS2, HR) received Interfant-21 followed by Blinatumomab (2 blocks). After remission allogenic HSCT (alloHSCT) was performed – complete remission (CR)1. A very early (VE) extramedullary relapse (EMR) was confirmed. 9-month boy (pre-B (11q23 MLL), CNS1) was treated with Interfant-06. After VE isolated EMR and IntRe-ALL-HR 2010 Blinatumomab was given (2 blocks) – CR2. Both infants had no Blinatumomab toxicity. 2-y.o. (pre-B , CNS1, HR), 3-y.o. (pre-B, CNS2, standard risk) treated with Nopho ALL-2008 presented with 1^st^ isolated early bone marrow relapse (BMR), Maintenance I and Maintenance II, respectively. 2-y.o. boy was resistant to IntReALL 2010 HR, post Blinatumomab measurable residual disease was 7%. FLAG (fludarabine, cytarabine, filgrastim), Bortezomib, Daratumumab were ineffective. 3-y.o. boy had inadequate response (IR) to IntReALL 2010 HR, after Blinatumomab (1 block) response was achieved, followed by alloHSCT. 2^nd^ isolated BMR was treated with chimeric antigen receptor T-cell therapy – IR. After Blinatumomab (2 blocks) remission allowed for alloHSCT – CR4. Both had no Blinatumomab toxicity. 14-y.o. girl (pre-B, CNS1, HR) after Nopho ALL-2008 had 1^st^ isolated VE BMR. No response to IntReALL 2010 HR, Blinatumomab (1 block). In this case Blinatumomab had neurotoxicity.

**Conclusions.:**

In Lithuania 2017–2021 4 R/R-ALL cases received Blinatumomab, 1 case as 1^st^ line ALL treatment. About half of cases had good response with minimum toxicity.

## References

[1] Brown PA, Ji L, Xu X, et al. Effect of Postreinduction Therapy Consolidation With Blinatumomab vs Chemotherapy on Disease-Free Survival in Children, Adolescents, and Young Adults With First Relapse of B-Cell Acute Lymphoblastic Leukemia: A Randomized Clinical Trial. JAMA. 2021;325(9):833–842. doi:10.1001/jama.2021.066933651090PMC7926290

[2] Queudeville M, Ebinger M. Blinatumomab in Pediatric Acute Lymphoblastic Leukemia—From Salvage to First Line Therapy (A Systematic Review). *Journal of Clinical Medicine*. 2021; 10(12):2544. 10.3390/jcm10122544.34201368PMC8230017

# Translation: Ifosfamide Induced Neurotoxicity in Soft Tissue Tumours

**Authors:** Kajus Merkevičius, Indrė Tamulienė, Vilma Rutkauskaitė

**Affiliations:** 1 Clinic of Pediatrics, Institute of Clinical Medicine, Faculty of Medicine, Vilnius University, Vilnius, Lithuania; 2 Center for Pediatric Oncology and Hematology, Vilnius University Hospital Santaros Klinikos, Vilnius, Lithuania

## Abstract

**Background.:**

In pediatric population Ifosfamide (IFO) is a widely used antineoplastic drug. IFO-induced encephalopathy (IIE) develops in 10-40% of adult patients, some studies report incidence up to 10% in children. Renal, liver function, drug interactions are studied as possible risk factors. IIE mostly requires symptomatic treatment, while methylene blue (MB) prophylaxis is investigated [1-3].

**Aim.:**

To study IIE in children with soft tissue tumours.

**Methods.:**

Retrospective case analysis.

**Results.:**

At tertiary centre 3 male patients were treated with IFO, 4 years old (y.o.) had retroperitoneal embryonic rhabdomyosarcoma, while 7 y.o., 8 y.o. had Ewing sarcomas. 7 y.o. presented with the most mild neurotoxicity symptoms (disorientation, agitation) that were present after just 5 IFO doses, on a 2^nd^ day of VIDE (block 2; Ewing 2008 protocol). In contrast, 8 y.o. was treated with the same protocol and had more distinct symptoms: vertigo, disorientation in surroundings, agitation with visual hallucinations after 19 IFO doses on 1^st^ VAI (block 1) day. In both cases IFO was discontinued, in the latter IFO was changed to Cyclophosphamide (CP), the 7 y.o. additionally received MB. In both cases neurological symptoms regressed. The most severe symptoms: vomiting and 10 minute seizure episode (confusion, dextral deviation of head, eyes, tonic contractions of the limbs), were present in the 4 y.o., electroencephalography results were concluded as IIE. Symptoms presented after 11 IFO doses on 1^st^ I^2^VA (block 6) day. IFO was changed to CP, additionally MB and diazepam were given – neurological symptoms regressed.

**Conclusions.:**

At Lithuanian tertiary centre treatment with IFO induced a variety of neurotoxicity symptoms at different treatment stages. A hypothesis that a trend of younger age and later onset of symptoms are related to a more sever neurotoxicity presentation could be raised. Further research on risk factors, prophylaxis, treatment and long-term consequences are needed.

## References

[1] Ide Y, et al. Analysis of the clinical characteristics of pediatric patients who experience ifosfamide-induced encephalopathy. Pediatr Blood Cancer. 2019 Dec;66(12):e27996. doi: 10.1002/pbc.27996. Epub 2019 Sep 18.31535455

[2] Sarbay H, et al. Ifosfamide induced encephalopathy in a child with osteosarcoma. J Oncol Pharm Pract. 2021 Jul;27(5):1302-1306. doi: 10.1177/1078155220963545. Epub 2020 Oct 6.33023384

[3] Vazirian F, et al. Aprepitant, fosaprepitant and risk of ifosfamide-induced neurotoxicity: a systematic review. Cancer Chemother Pharmacol 90, 1–6 (2022). 10.1007/s00280-022-04439-x35635561

# Translation: Haematopoietic Stem Cell Transplantation in Primary Immunodeficiencies in Lithuania

**Authors:** Audronė Mulevičienė, Ramunė Vaišnorė, Ramunė Pasaulienė, Ignė Kairienė, Audronė Jakaitienė, Goda Vaitkevičienė, Jelena Rascon

**Affiliations:** 1 Centre for Paediatric Oncology and Haematology, Vilnius University Hospital Santaros Klinikos, Vilnius, Lithuania; 2 Institute of Data Science and Digital Technologies, Vilnius University, Lithuania; 3 Vilnius University, Faculty of Medicine, Lithuania

## Abstract

**Background.:**

Primary immunodeficiencies (PIDs) are heterogeneous group of inborn errors of immunity. The most severe forms are life-threatening and require haematopoietic stem cell transplantation (HSCT) for curative effect. The aim of our study was to evaluate the outcomes of HSCT performed in children with PIDs in Centre for Paediatric Oncology and Haematology, Vilnius University Hospital Santaros Klinikos during 2010-2021 year period.

**Methods.:**

Medical records were retrieved and a retrospective analysis was carried out.

**Results.:**

Since 2010, when the first HSCT was performed for this patient group in Lithuania, 16 children (11 males and 5 females) underwent HSCT due to these conditions: severe combined immune deficiencies (n = 9), Wiskott-Aldrich syndrome (n = 1), chronic granulomatous disease (n = 2), haemophagocytic lymphohistiocytosis (n = 3), immune deficiency of unknown genetic variant (n = 1). Median age of the patients was 1.11 years (95% IQR 0.60-7.11). In total, five (31%) were transplanted from matched sibling donor (MSD) and 11 (69%) from matched unrelated donor (MUD). Median duration of follow-up after HSCT was 4.15 years (95% CI [3.27, 6.37]). At 3 years overall survival according to the donor type MSD vs MUD was 1.000 (95% CI [1.000, 1.000]) vs 0.623 (95% CI [0. 0.389, 0.999]), p = 0.1366, event free survival was 1.000 (95% CI [1.000, 1.000]) vs 0.455 (95% CI [0.238, 0.868]), p = 0.0558.

**Conclusions.:**

The outcomes of HSCT in children diagnosed with PIDs in our centre during 2010-2021 years period are encouraging and correspond to the results reported by main HSCT centres. The best outcomes were seen in children transplanted from a MSD.

# Translation: HSCT for Latvian Children - from Early-Stage Salvage option to regular therapeutic alliance with a Lithuanian Center

**Authors:** Zanna Kovalova, Anda Kivite-Urtane, Elizabete Cebura, Marika Grutupa, Anna Valaine, Zelma Vishnevska-Preciniece, Jelena Rascon

**Affiliations:** 1 Department of Hematology and oncology, Children’s Clinical University Hospital, riga, Latvia;; 2 Department of Paediatrics, riga Stradins University, riga, Latvia; 3 Institute of Public Health, riga Stradins University, Latvia; 4 Department of Public Health and epidemiology, riga Stradins University, Latvia; 5 Center for Pediatric oncology and Hematology, Vilnius University Hospital Santaros Klinikos, Vilnius, Lithuania; 6 Vilnius University, Faculty of Medicine

## Abstract

**Background:**

Hematopoietic stem cell transplantation (HSCT) became a clinically realistic and financially supported option for Latvian children in 2005 when Latvia joined the eU.

**Aim of the study:**

Clinical data of transplanted Latvian children with different diagnoses were retrospectively analyzed to evaluate outcomes during different periods and at different transplantation centers.

**Patients and Methods:**

30 consecutive patients after alloHSCT were enrolled during the „early“ transplantation phase (2005-2018) in Germany/Hungary/Sweden in the proportion of 25/4/1 respectively. During the Lithuanian period (2011-2022), 31 patients were enrolled for analyses including auto- (n=9) and alloHSCT (n=22). Descriptive analysis was performed, and Kaplan-Meier survival curves were constructed. results were considered statistically significant if p<0.05.

**Results:**

Clinical characteristics of both patient groups are statistically different in distribution by diagnosis and by indications for transplantation (p=0.025). the Median follow-up time in both groups was 39.0 months (interquartile range (IQr) 13.5-80.5). Median age at transplantation and sex distribution were similar in both groups (p=0.36 and p=0.94 respectively). the median interval from diagnosis to transplantation was 18.5 (IQr 7.0-33.0) months during the „early phase“ and 11.0 (IQr 7.0-24.0) for patients, transplanted during the Lithuanian phase (p=0.53). Despite apparent differences in 12-month survival proportions between groups (71.4% for LT phase vs. 57.1% for „early phase“), no significant differences were found between the overall survival curves (p=0.95).

**Conclusion:**

There is still room for further improvement for the whole healthcare team of both countries including a uniform approach for indications for HSCT, usage of the same therapeutic programs, and certified laboratory facilities.

# Translation: Outcomes of Pediatric Hematopoietic Stem Cell Transplantation in Lithuania

**Authors:** Jelena Rascon, Ramunė Vaišnorė, Ramunė Pasaulienė, Zhanna Kovalova, Audronė Jakaitienė, Goda Vaitkevičienė

**Affiliations:** 1 Center for Pediatric Oncology and Hematology, Vilnius University Hospital Santaros Klinikos, Vilnius, Lithuania; 2 Vilnius University, Faculty of Medicine; 3 Institute of Data Science and Digital Technologies, Vilnius University, Vilnius, Lithuania; 5 Department of Hematology and Oncology, Children‘s Clinical University Hospital, Riga, Latvia;; 5 Riga Stradins University, Riga, Latvia

## Abstract

**Background.:**

The pediatric hematopoietic stem cell transplantation (HSCT) program at VUHSK was launched in 2002. It provides autologous and allogeneic services for all Lithuanian and, since 2011, Latvian children.

**Aims.:**

We aimed to assess change in HSCT outcomes over two decades.

**Methods.:**

A retrospective analysis of all consecutive HSCTs from 2002 to 2021 was performed. Two time periods (2002-2011 and 2012-2021) were compared to evaluate the change. The 5-year overall survival (OS_5y_), the cumulative incidence of relapse (CIR) in the malignant setting and transplant-related mortality (TRM) were analyzed. Descriptive statistics and Kaplan-Meier survival estimates were calculated.

**Results.:**

From 2002 to 2021 totally 269 HSCTs (56.5% (152/269) allogeneic and 43.5% (117/269) autologous) were performed in 246 unique recipients. The median annual number of allogeneic HSCT increased from 6 in 2002-2011 to 11 in 2012-2021. Non-Lithuanian citizens comprised 22.2% of all unique recipients (32/144) in 2012-2021.

In the autologous setting, no changes occurred comparing OS_5y_, CIR and TRM in 2002-2011 (n=52/103) vs 2012-2021 (n=51/103).

In the allogeneic setting a significant improvement in OS_5y_ from 0.380 (95% CI 0.267, 0.541) in 2002-2011 (n=50/144) to 0.768 (95% CI 0.686, 0.861) in 2012-2021 (n=93/144), p < 0.0001, was observed. The TRM at 100 days decreased from 0.260 (95% CI 0.143, 0.380) in 2002-2011 to 0.099 (95% CI 0.046, 0.172) in 2012-2021, p <0.0001 and at 5 years – from 0.507 (95% CI 0.353, 0.643) to 0.165 (95% CI 0.097, 0.249), p < 0.0001, respectively. None of the patients transplanted from an HLA-identical sibling in 2012-2021 (n=25) succumbed due to TRM.

The subgroup analysis of 85 patients transplanted for malignant diseases showed a significant decrease in the CIR_5y_ from 0.435 (95% CI 0.213, 0.639) in 2002-2011 (n=34/85) to 0.156 (95% CI 0.062, 0.290) in 2012-2021 (n=51/85), p = 0.043.

**Conclusions.:**

Despite a small transplant volume, a significant improvement over two decades was demonstrated. A close collaboration between Lithuania and Latvia and centralized patient referral is essential to maintain sufficient transplant volume and ensure safe transplant quality.

# Translation: Assessment of Fertility Care in Childhood Cancer Patients

**Authors:** Eglė Stukaitė-Ruibienė, M.E.M. van der Perk, Goda Elizabeta Vaitkevičienė, Žana Bumbulienė, M.M. van den Heuvel-Eibrink, Jelena Rascon

**Affiliations:** 1 Vilnius University, Faculty of Medicine, Vilnius, Lithuania; 2 Princess Máxima Center for Pediatric Oncology, Utrecht, Netherlands; 3 Center for Pediatric Oncology and Hematology, Vilnius University Hospital Santaros Klinikos, Vilnius, Lithuania; 4 Center of Obstetrics and Gynecology, Vilnius University Hospital Santaros Klinikos, Vilnius, Lithuania

**Keywords:** Childhood cancer, fertility counseling, late effects, questionnaire, reproductive health

## Abstract

**Background.:**

A five-year survival rate of childhood cancer exceeds 80%. However, most survivors develop late effects including infertility. It is recommended to discuss fertility issues before the start of treatment. However, most survivors perceive the information on fertility as insufficient. The aim of this study was to assess the experience of the fertility care (FC) in childhood cancer patients (CCP) within the framework of the EU-Horizon 2020 TREL project.

**Material and Methods.:**

All parents of CCP and patients aged 12-17.9 years were invited to complete validated oncofertility-care-questionnaires. A triage system was developed to stratify patients according to their infertility risk. Demographic and treatment data was retrieved from medical records and assessed using descriptive statistics, a risk of gonadal damage was evaluated using the triage.

**Results.:**

Forty-four of 86 distributed questionnaires (51.2%) were completed. A majority (26, 59.1%) of respondents was exposed to low, 18 (40.9%) – to high risk of gonadal damage. Most (15, 83.3%) high infertility risk respondents were not counseled by a fertility specialist (FS). Four boys were counseled. More than half (27, 61.4%) of respondents did not know their infertility risk. Only 4 (10.5%) respondents informed on fertility by a pediatric oncologist received supportive material (vs 75% counseled by a FS), 11 (28.9%) had to ask information on fertility themselves (vs 0%), 19 (50%) still had questions about fertililty (vs 0%). All respondents counseled by a FS thought they know enough about fertility (vs 14, 37.8%), and all knew fertility preservation options (vs 13, 35.1%).

**Conclusions.:**

A majority of high infertility risk respondents was not counseled by a FS. Respondents counseled by a FS perceived the quality of FC better than those informed by a pediatric oncologist only. FC should be improved, thus the patients should be triaged on their risk of gonadal damage at the moment of diagnosis.

# Translation: Refractory ALL Despite the Use of Innovative Therapies

**Authors:** Jelena Rascon, Goda Vaitkevičienė, Vilma Rutkauskaitė, Jochen Büchner

**Affiliations:** 1 Center for Pediatric Oncology and Hematology, Vilnius University Hospital Santaros Klinikos, Vilnius, Lithuania; 2 Vilnius University, Faculty of Medicine; 1 Oslo University Hospital, Norway

## Abstract

**Background.:**

Innovative therapies offer a cure for relapsed/refractory hematologic malignancies. However, disease biology remains of paramount importance for survival. We aim to present a case of resistant acute lymphoblastic leukemia (ALL) despite the use of innovative approaches.

**Case description.:**

The boy was diagnosed with ALL, CNS1 at 3 years of age. The leukemic population expressed a B-precursor phenotype, hyperdiploid karyotype (50XY) without genetic aberrations. The patient was treated according to the NOPHO-ALL-2008 protocol, achieved 1CR, negative MRD (MRD-), and was stratified to the standard risk group. Still on maintenance therapy, the 1^st^ early bone marrow (BM) relapse, CNS2 was diagnosed. Two courses of high-risk chemotherapy failed to achieve remission. Thus, blinatumomab was administered and 2CR/MRD-was documented. The patient was consolidated with allo-HSCT from HLA-identical sibling 2 years and a half after the initial diagnosis. A myeloablative chemotherapy-based conditioning was used prior to PBSC infusion. The post-transplant course was uneventful except for mild chronic GvHD. However, 11 months after HSCT the 2nd BM relapse, CNS2 developed. Relapsed leukemic cells expressed identical diagnostic phenotype, but karyotype turned from hyper- to normoploid (46YX). No genetic aberration was detected either. The patient was referred to Oslo University Hospital where CAR-T cells (CART19, Kymriah©) were infused. The post-infusion toxicity was grade 0-I. Thereafter the 3CR/ MRD-was documented, however, continued only for 2 months. Due to increasing MRD the 2^nd^ CAR-T cell dose of the same product was infused, unfortunately with no effect – the 3^rd^ BM relapse was diagnosed in 1 month after the 2^nd^ dose. Two courses of blinatumomab induced the 4CR/MRD-, consolidated with the 2^nd^ allo-HSCT from the same donor after TBI. Despite all efforts increasing MRD was documented 6 months after HSCT, nearly 5 years after initial diagnosis.

**Conclusion.:**

A better understanding of ALL biology is needed to overcome resistant disease and offer personalized therapy.

# Translation: First Experience of NTRK Sarcoma in Lithuania: from Challenging Diagnosis to Targeted Therapy

**Authors:** Vaiva Čenkutė, Ryte Damaševičiūtė, Dmitrij Šeinin, Irina Adomaitienė, Julija Ravinskienė, Sonata Saulytė Trakymienė

**Affiliations:** 1 Faculty of Medicine, Vilnius University, Lithuania; 2 National Centre of Pathology; 3 Vilnius University Hospital Santaros Clinics; 4 Clinic of Children’s Diseases, Faculty of Medicine, Vilnius University, Vilnius University Hospital Santaros Clinics, Vilnius, Lithuania

## Abstract

**Introduction:**

Neurotrophic tyrosine receptor kinase (NTRK) gene fusions are drivers initiating oncogenic pathways in various tumors. Tropomyosin-related kinase (TRK) inhibitors are highly selective for the TRK family receptors. After the discovery of NTRK gene fusions, the treatment has become more personalized and effective by usage of targeted therapy. One of the orally administered drugs, which shows a promising efficacy in LMNA-NTRK1 soft-tissue sarcomas is Larotrectinib.

**Case description:**

A 9-year-old girl presented with a distinct mass on her left thigh, posteriorly above popliteal region and 5-6 kg loss of weight within 6 months. Patient also complained of the pain in her left thigh, more intense at night, and dry cough. Instrumental examination showed locally advanced large tumor (69x54x115mm) of the thigh without signs of overgrowth into the femur, but with multiple metastasis in both thighs and calves, and multiple pulmonary metastases with pleural involvement. Performed biopsy indicated a low-grade infantile fibrosarcoma-like neoplasm, which was specified to be LMNA-NTRK1 subtype. Complete surgical resection was not possible, however surgery of a metastatic mass from the left thigh was performed due to patient’s complaints of pain. Within one month after the diagnosis, no signs of tumour progression was observed on whole body MRI, thus patient was administered oral tyrosine receptor kinase (TRK) inhibitor Larotrectinib 100 mg twice daily. Within 3 months TRK inhibitor led to a remarkable response: disappearance of dry cough, significant decrease of primary tumour and near-complete resolution of metastases in both legs and lungs. At 12 months of targeted therapy, the patient retains response (stable disease) and favorable safety.

**Conclusions:**

This report describes a first type of NTRK-rearranged paediatric soft tissue sarcoma in Lithuania. Testing for NTRK gene fusions in paediatric tumours with nonspecific morphology is highly important because of the recent availability of targeted therapy. The efficacy of Larotrectinib has been demonstrated to be rapid and durable with various tumour types, including soft-tissue sarcomas. Our experience treating the patient with Larotrectinib showed a positive response with minimal toxicity. Personalized therapy allows controlling the disease, when conventional treatment could not be applied.
